# Supplementary material for: A SUMO-dependent feedback loop senses and controls the biogenesis of nuclear pore subunits
Source: Nat Commun. 2018 Apr 25;9:1665. doi: 10.1038/s41467-018-03673-3 (PMC5916902; doi:10.1038/s41467-018-03673-3)
Supplement: Supplementary file 1 — Supplementary Information(PDF 25441 kb) [file 41467_2018_3673_MOESM1_ESM.pdf]

<u>SUPPLEMENTARY INFORMATION</u>

A SUMO-dependent feedback loop senses and controls the biogenesis of nuclear pore subunits

Rouvière et al.

<u>**Supplementary Information** includes:</u>

Supplementary Figures 1-5

Supplementary Table 1: Yeast strains used in this study
Supplementary Table 2: Plasmids used in this study
Supplementary Table 3: qPCR primers used in this study

Supplementary References

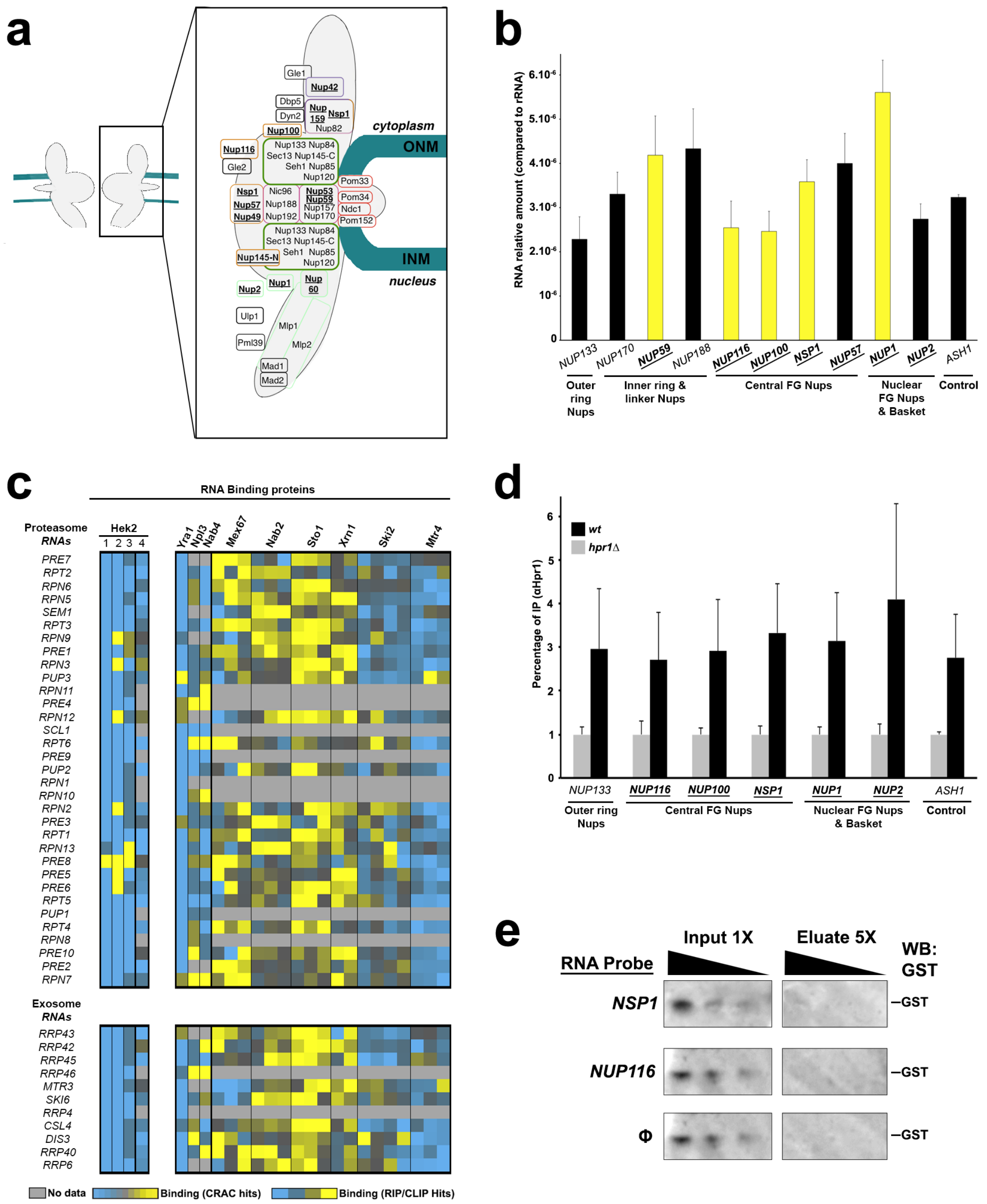

Supplementary Figure 1. The association of Hek2 to a subset of *NPC* mRNAs is specific.

a, Schematic representation of the yeast nuclear pore complex indicating the relative position of each nucleoporin within subcomplexes or along the NPC axis. FG-Nups appear in bold, underlined. ONM, outer nuclear membrane. INM, inner nuclear membrane. **b,** *NPC* mRNAs levels (mean ± SD; n=3; relative to rRNA) were measured by RT-qPCR in *HEK2-pA* strains. Hek2-bound *NPC* mRNAs appear in yellow. **c,** RBP binding was analyzed as in **Fig. 1a** for mRNAs encoding proteasome or exosome subunits. **d**, Hpr1-associated mRNAs were immunopurified using anti-Hpr1 antibodies[1] and quantified by RT-qPCR using specific primer pairs. Percentages of IP (mean ± SD; n=3) are the ratios between purified and input RNAs, set to 1 for *hpr1Δ* control cells. **e**, Recombinant GST was incubated with streptavidin beads either naïve (Φ) or previously coated with biotinylated RNA probes encompassing Hek2-binding sites from *NSP1* or *NUP116*. Decreasing amounts of input and eluate fractions were loaded to allow comparison as in **Fig. 1e**.

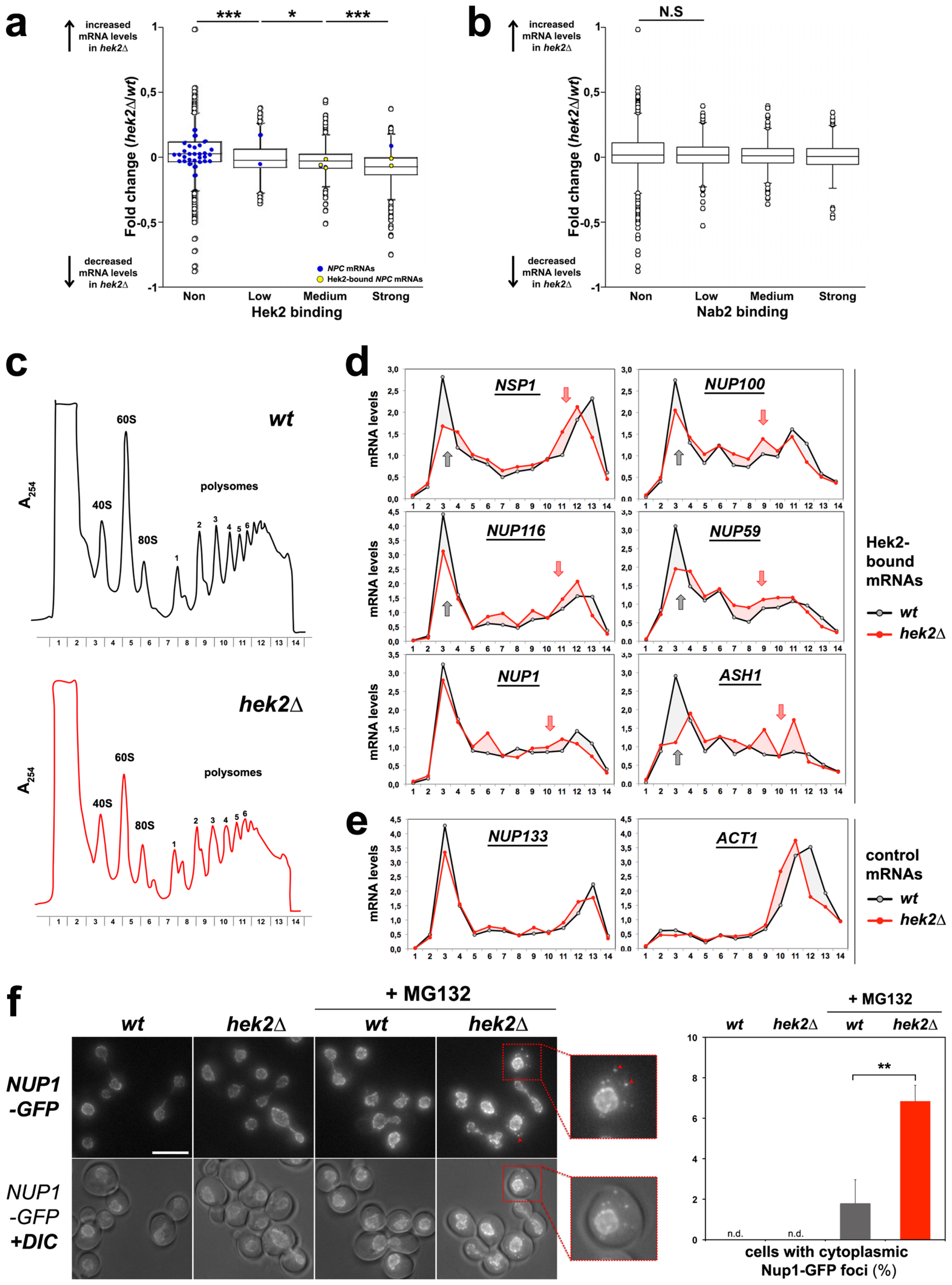

Supplementary Figure 2 *(see legend on next page).*

Supplementary Figure 2. Hek2 binding does not affect the levels of *NPC* mRNA but rather modulates their ribosome occupancy.

a, mRNAs were split in four categories depending on their binding to Hek2[2]. For each group of transcripts, the averaged log2 of the *hek2Δ/wt* ratios calculated from two independent microarray hybridizations were plotted. Boxplots were generated using KaleidaGraph (Synergy Software): each box encloses 50% of the values, the median is displayed as a line, and the bars extending from the top and bottom of each box mark the minimum and maximum values within the dataset falling within an acceptable range. Values falling outside of this range are displayed as individual points. mRNAs encoding NPCs components are highlighted in two different colors depending on their association to Hek2. Note that mRNAs strongly bound by Hek2 tend to be less abundant in the absence of this protein. * $P<0.5$; ***$P<0.001$ (Mann-Whitney-Wilcoxon test). **b,** The same analysis as in **a.** was performed after grouping the transcripts according to their binding to Nab2[3]. Note the absence of correlation between Nab2 binding and the changes in mRNA levels scored upon *HEK2* inactivation. N.S, not significant. **c,** Polysome fractionation from *wt* and *hek2Δ* cells from the BY4742 background. The absorbance at 254nm (A_{254}) recorded during the collection of the different fractions of the sucrose gradient is displayed. The positions of 40S, 60S, 80S ribosomal species and polysomes are indicated, as well as the number of ribosomes per mRNA in the polysomes fractions. Note that polysome profiles from these *hek2Δ* mutant cells exhibit reproducible discontinuities typical of half-mer formation, i.e. polysomes lacking stoichiometric amounts of both 60S and 40S ribosomal subunits. While this phenotype could reflect impaired 60S biogenesis, defective coupling of 60S subunits to 40S-mRNA complexes or general translational derepression[4,5,6], it was not observed in *hek2Δ* mutant cells of an alternate genetic background (W303, **Fig. 2c**), suggesting that it is not solely caused by *HEK2* inactivation. **d**, Relative distribution of the *NSP1*, *NUP100*, *NUP116*, *NUP59*, *NUP1* and *ASH1* mRNAs in polysome gradients from the same *wt* (black lines) and *hekΔ* (red lines) cells. mRNAs amounts in each fraction were quantified by RT-qPCR, normalized to the sum of the fractions and to the distribution of a control spike RNA. Grey arrows indicate a decrease in the amounts of mRNAs found in the light fractions in *hek2Δ* cells. Red arrows point to an increase in the quantity of mRNAs found in the polysomes fractions of the mutant. These results are representative of four independent experiments (two performed in the W303 background, two in the BY4742 background; see **Fig. 2**). **e,** Same as **d,** for *NUP133* and *ACT1* control mRNAs. **f,** *Left panel,* Fluorescence microscopy analysis of drug-responsive (*erg6Δ*) derivatives of *wt* and *hek2Δ* cells expressing a GFP-tagged version of Nup1, and treated with MG132 for 2h at 30°C. Images of single-channel fluorescence for GFP are shown (*top row*), as well as overlay images with DIC (differential interference contrast, *bottom row*), and 2-fold magnifications of a MG132-treated *hek2Δ* cell exhibiting cytoplasmic Nup1 foci. Scale bar, 5 μm. *Right panel*, quantification of the number of cells exhibiting cytoplasmic Nup1 foci (mean ± SD; n=3; at least 100 cells counted per category and experiment). n.d., not detectable. ** $P<0.01$ (Welch's t-test).

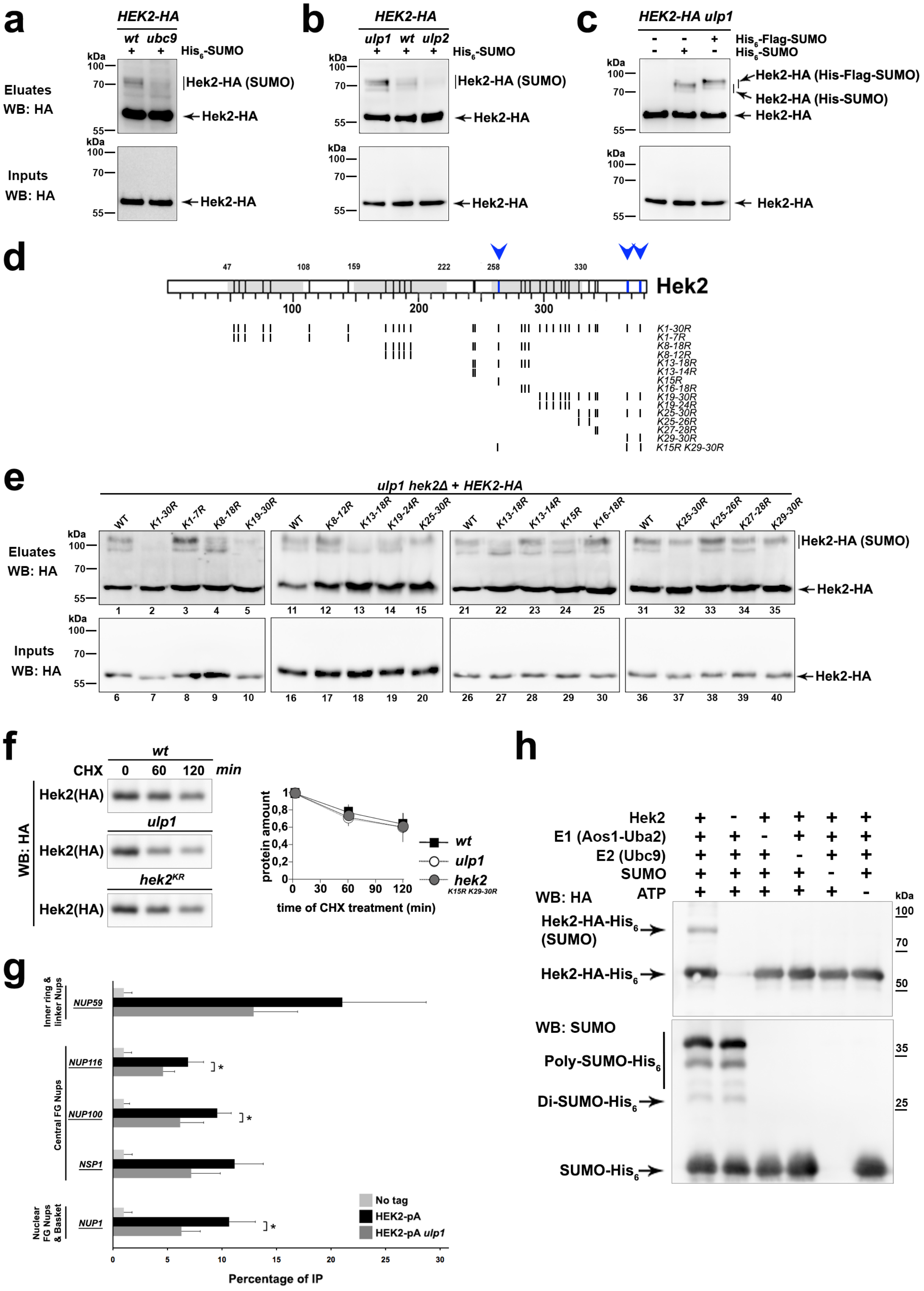

Supplementary Figure 3 *(see legend on next page).*

Supplementary Figure 3. Characterization of Hek2 sumoylation.

a-c, Extracts from *HEK2-HA* and *HEK2-HA ubc9* cells (**a**), *HEK2-HA, HEK2-HA ulp1* and *HEK2-HA ulp2Δ* cells (**b**), or *HEK2-HA ulp1* cells expressing the indicated His-SUMO constructs (**c**) were used for nickel chromatography. Total lysates ("Inputs") and purified His-SUMO conjugates ("Eluates") were analyzed by western blotting using anti-HA antibodies. The positions of the sumoylated and unmodified versions of Hek2-HA, as well as molecular weights, are indicated. *ubc9* mutant cells carry the *ubc9-1* thermosensitive allele which destabilizes the Ubc9 protein at restrictive temperature, abolishing cellular sumoylation[7]. Note that the decreased Hek2 sumoylation scored in *ulp2Δ* cells is likely caused by the reduced availability of conjugatable SUMO previously observed in this mutant[8]. **d**, Schematic representation of the Hek2 protein and of the different *KR* mutants used in this study. Each vertical bar corresponds to a lysine residue and the KH-domains are displayed in grey, together with their boundaries as small numbers. For *KR* mutants, vertical bars represent the lysines that were mutated into arginines. The sumoylated residues identified in this study are indicated by blue bars and arrowheads. **e**, Hek2 sumoylation was analyzed in the indicated *KR* mutants as in **a-c**. Total lysates ("Inputs", *bottom panel*) and purified SUMO-conjugates ("Eluates", *top panel*) were analyzed by immunoblotting with anti-HA antibodies. The positions of the sumoylated and unmodified versions of Hek2-HA, as well as molecular weights, are indicated. **f**, Protein levels of HA-tagged versions of Hek2 were evaluated in *wt*, *ulp1* and *hek2 K15R K29-30R* (*hek2^{KR}*) cells treated with cycloheximide (CHX) for the indicated time (minutes). Whole cell extracts were analyzed by western blotting using anti-HA antibody. The relative amounts of Hek2-HA (mean ± SD; n=2) were quantified over the time following CHX treatment and are expressed relative to t=0. **g**, Hek2-pA-associated mRNAs were immunopurified and quantified from *wt* ("no tag"), *HEK2-pA* and *HEK2-pA ulp1* cells as in **Fig. 1b**. Percentages of IP (mean ± SD; n=3) are the ratios between purified and input RNAs, further normalized to the amount of purified bait and set to 1 for the "no tag". Values for *wt* are the same as used in **Fig. 1b**. **h**, *In vitro* sumoylation of recombinant Hek2 was performed in the presence (+) or the absence (-) of the indicated components and the reactions were analyzed by western blotting using anti-HA and anti-SUMO antibodies. The positions of the sumoylated and unmodified versions of Hek2, of different poly-SUMO chains and of molecular weights are indicated. Note that the modified version of Hek2 is only detectable upon incubation of the recombinant protein with the unique combination of purified E1, E2, SUMO and ATP.

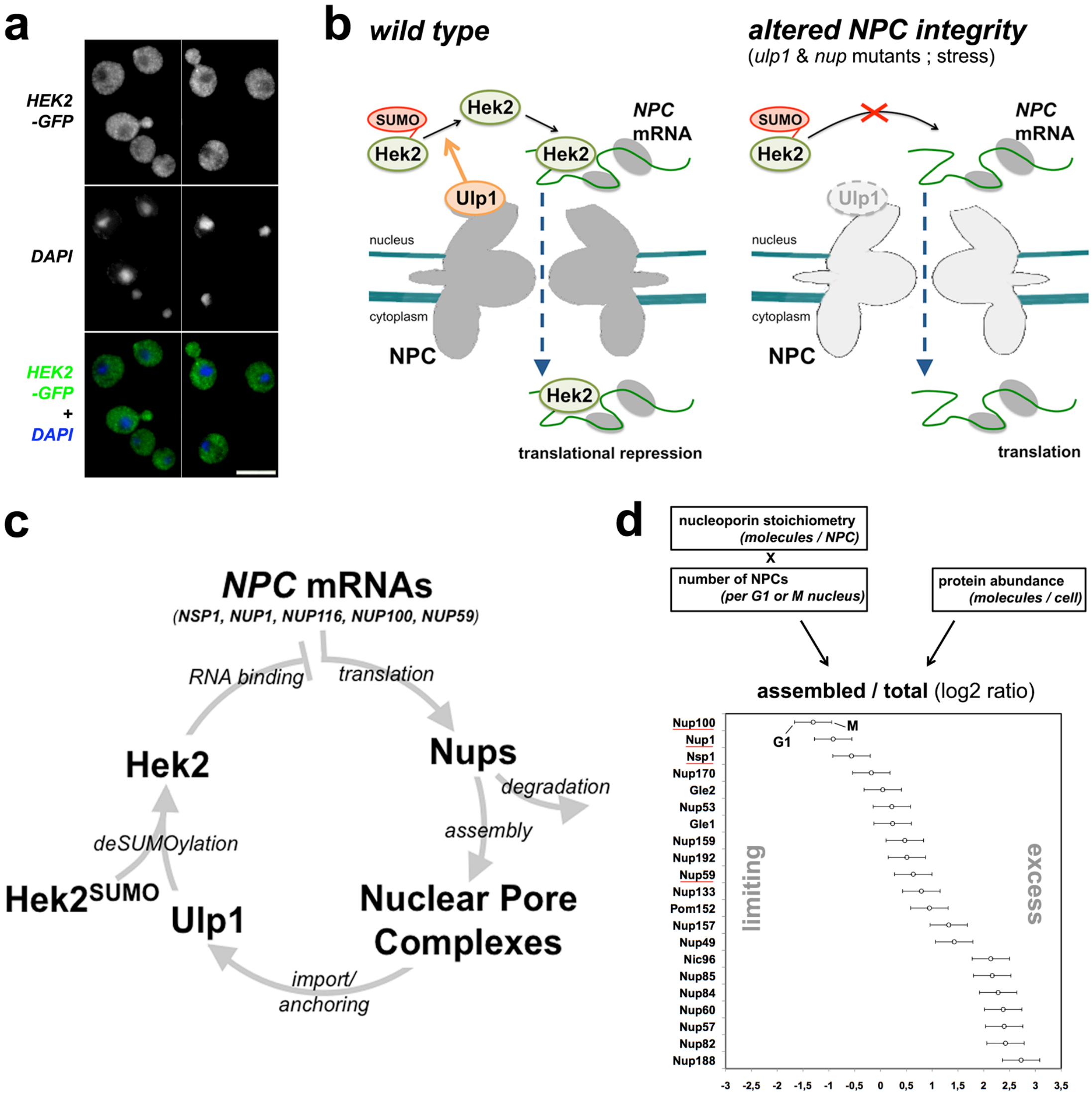

Supplementary Figure 4. A SUMO-dependent feedback loop regulates the availability of nucleoporins.

a, Fluorescence microscopy analysis of *HEK2-GFP* cells. Nuclei were stained with DAPI following fixation. Images of single-channel fluorescence for GFP and DAPI are shown, as well as overlay images. Scale bar, 5 μm. Note that Hek2 is predominantly localized in the cytoplasm. **b,** Schematic representation of the relationships between Ulp1 activity, Hek2 function and *NPC* mRNA expression scored in this study. In conditions of altered NPC integrity (right panel), decreased Ulp1 stability leads to the accumulation of sumoylated, inactive versions of Hek2, potentially releasing *NPC* mRNAs from their translationally-repressed state. **c,** Model for a feedback loop involving Ulp1 as a sensor of NPC integrity and controlling nucleoporin homeostasis through Hek2-mediated translational repression. **d,** For each nucleoporin, the amounts of proteins expected to be assembled in NPCs were calculated by multiplying the empiric values for NPC stoichiometry[9] by the total numbers of NPCs per nucleus, as counted in G1 or M cells[10]. These values were further divided by the total cellular amounts of nucleoporins (as experimentally determined)[11] and the log2 of these ratios were displayed. Horizontal bars reflect the expected variation between the G1 and M phases of the cell cycle. The more the displayed values are elevated, the more the corresponding nucleoporins are expected to be in excess as compared to the actual number of NPCs. Nucleoporins whose mRNAs are regulated by Hek2 are underlined in red. Note that this analysis does not include the nucleoporins that are part of other cellular complexes (i.e. Ndc1, Sec13, Seh1) or those for which abundances data were not available (Nup116, Nup145, Nup120, Nup2, Nup42, Pom34).

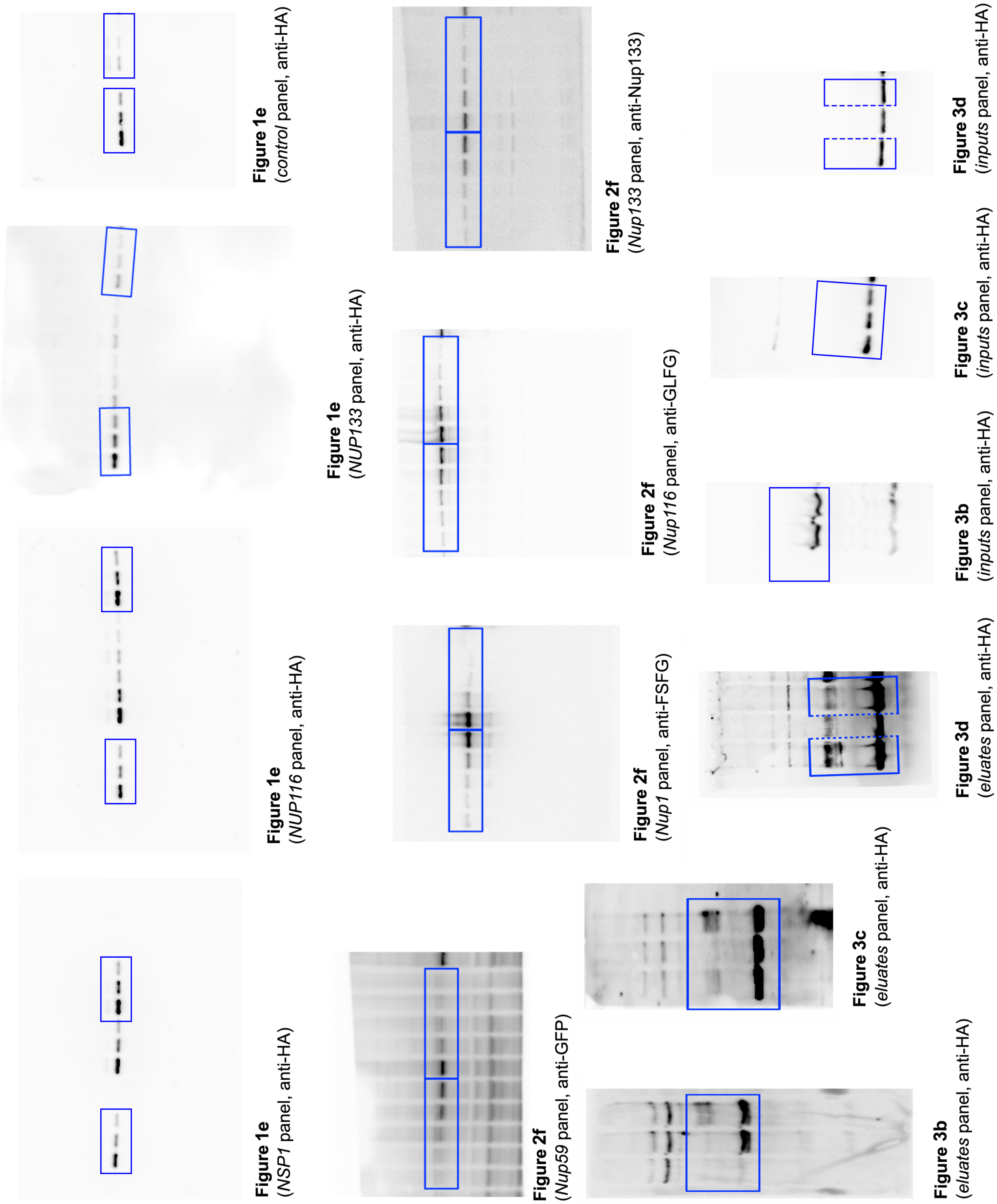

Supplementary Figure 5. Uncropped scans of the blot images shown in Figures.

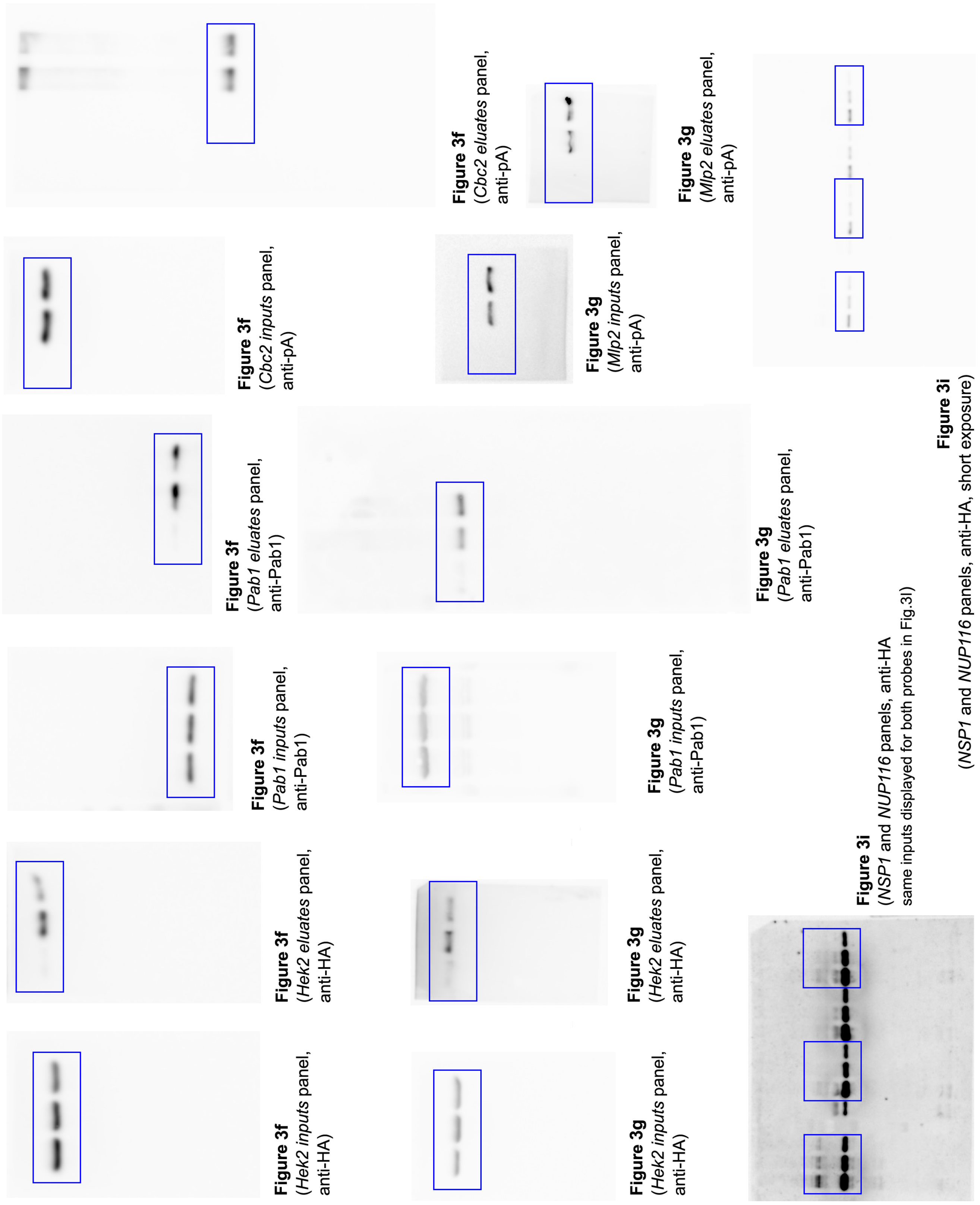

Supplementary Figure 5. Uncropped scans of the blot images shown in Figures (continued)**.**

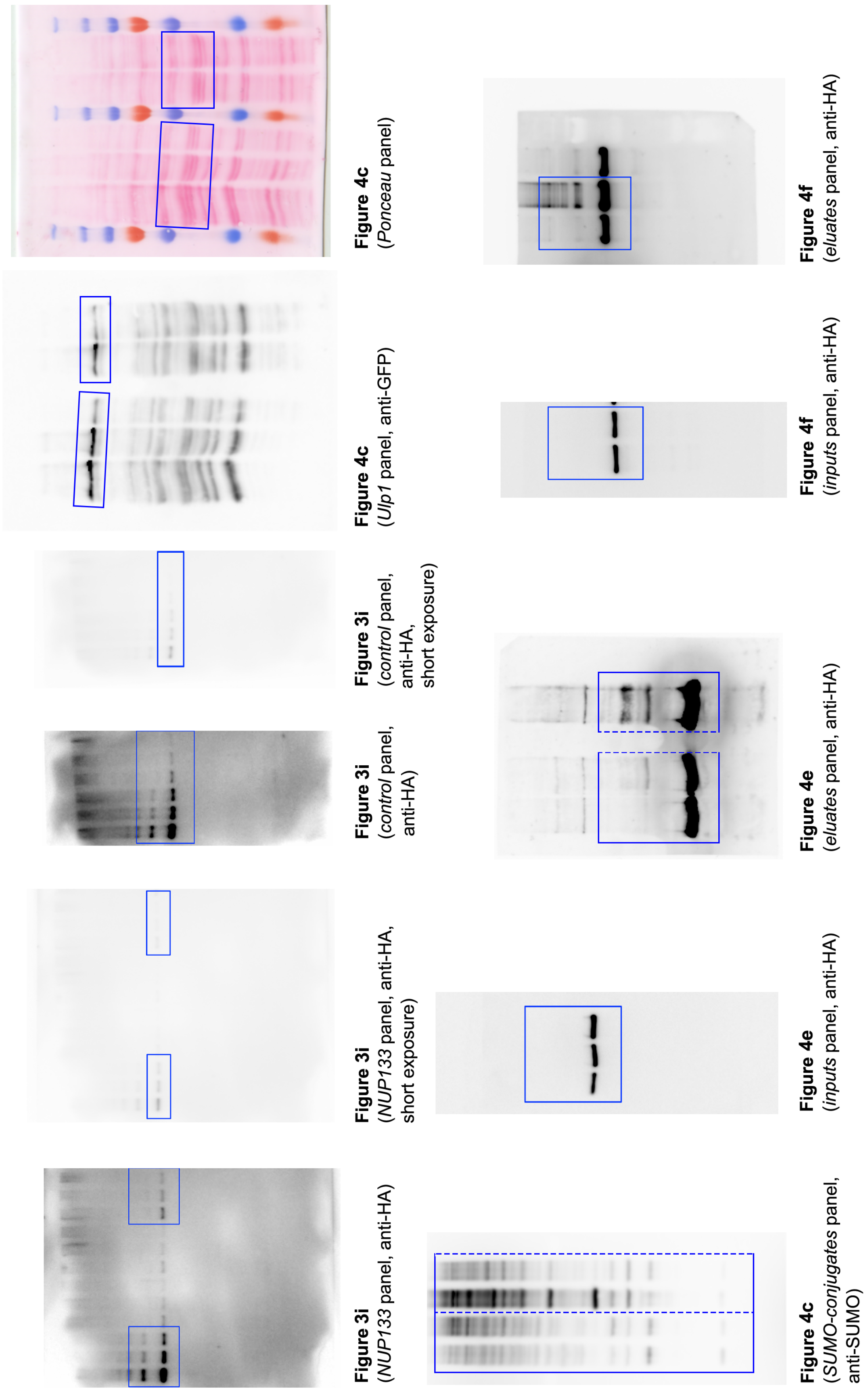

Supplementary Figure 5. Uncropped scans of the blot images shown in Figures (continued)**.**

Supplementary Table 1: Yeast strains used in this study

Strain code	Name	Relevant genotype	Source/Reference
BY4742 / BY4741	*wt*		Euroscarf
Y13058	*hek2Δ*	*hek2::kanMX*	Euroscarf
JR154	*HEK2-pA*	*hek2::kanMX pRS316-HEK2-protA*	This study *
Y14072	*hpr1Δ*	*hpr1::kanMX*	Euroscarf
K5552	*wt*	***(W303)*** *ASH1-MYC9*	12
YV1862	*hek2Δ*	***(W303)*** *ASH1-MYC9 hek2::kanMX*	This study (a)
YV2083	*NUP59-GFP*	*NUP59-GFP::HIS3MX*	Invitrogen
YV2084	*NUP59-GFP hek2Δ*	*NUP59-GFP::HIS3MX hek2::kanMX*	This study *
YV2056	*NUP1-GFP*	*NUP1-GFP::HIS3MX*	Invitrogen
Y10568	*erg6Δ*	*erg6::kanMX*	Euroscarf
YV2092	*NUP1-GFP erg6Δ*	*NUP1-GFP::HIS3MX erg6::kanMX*	This study *
YV2093	*NUP1-GFP erg6Δ hek2Δ*	*NUP1-GFP::HIS3MX erg6 ::kanMX hek2::kanMX*	This study *
YV1593	*HEK2-3HA*	*HEK2-3HA::kanMX*	This study (b)
YV1262	*ulp1*	*ulp1::kanMX YCpLac111-ulp1-333 (LEU2)*	1, 13
YV1432 / YV1433	*ulp1*	*ulp1::kanMX YCpLac22-HIS3-ulp1-333 (HIS3)*	This study (c)
YV1410	*ubc9-1*	*ubc9 ::kanMX pRS315-ubc9-1*	This study (d)
YV1664	*HEK2-3HA ubc9-1*	*HEK2-3HA::kanMX ubc9 ::kanMX pRS315-ubc9-1*	This study *
YV1626	*HEK2-3HA ulp1*	*HEK2-3HA::kanMX ulp1::kanMX YCpLac22-HIS3-ulp1-333 (HIS3)*	This study *
YV1076	*HEK2-HA*	***(W303)*** *pRS316-HEK2-3HA*	a gift from X. Zhao
YV1769	*HEK2-HA ulp1*	***(W303)*** *ulp1::HIS3 YCpLac22-ulp1-333 (TRP1) pRS316-HEK2-3HA (URA3)*	13
YV1078	*HEK2-HA ulp2Δ*	***(W303)*** *ulp2::kanMX pRS316-HEK2-3HA (URA3)*	a gift from X. Zhao
YV1171	*SMT3 shuffle*	*smt3::kanMX pRS316-SMT3 (URA3)*	This study (e)
YV1440 HEK2-HA	*HEK2-3HA ulp1 His-Flag-SMT3*	*smt3::kanMX ulp1::kanMX YCpLac22-HIS3-ulp1-333 (HIS3) pYES2-LEU2-His-Flag-SMT3 (LEU2) pRS316-HEK2-3HA (URA3)*	This study (f)
YV1668	*hek2Δ ulp1*	*hek2::kanMX ulp1::kanMX YCpLac22-HIS3-ulp1-333 (HIS3)*	This study *
JR274	*HEK2(K15R K29-30R)-3HA ulp1*	*hek2::kanMX ulp1::kanMX YCpLac22-HIS3-ulp1-333 (HIS3) pRS316-HEK2(K15R K29-30R)-3HA (URA3)*	This study *
YV1451	*CBC2-pA ulp1*	*CBC2-ProtA::His3 ulp1::kanMX YCpLac111-ulp1-333 (LEU2)*	1
YV1479	*MLP2-pA*	*MLP2-ProtA::HIS3*	1
YV1606	*HEK2-3HA CBC2-pA*	*HEK2-3HA::kanMX CBC2-protA::HIS3*	This study *
YV1601	*HEK2-3HA CBC2-pA ulp1*	*HEK2-3HA::kanMX CBC2-protA::HIS3 ulp1::kanMX YCpLac111-ulp1-333 (LEU2)*	This study *
YV1756	*HEK2-3HA MLP2-pA*	*HEK2-3HA::kanMX MLP2-protA::HIS3*	This study *
YV1757	*HEK2-3HA MLP2-pA ulp1*	*HEK2-3HA::kanMX MLP2-protA::HIS3 ulp1::kanMX YCpLac22-HIS3-ulp1-333 (HIS3)*	This study *
JR153	*HEK2-pA ulp1*	*hek2::kanMX ulp1::kanMX YCpLac22-HIS3-ulp1-333 (HIS3) pRS316-HEK2-protA*	This study *
SWY518	*wt*	***(W303)***	14
SWY2950	*nup145ΔFG nup57ΔFG nup100ΔFG*	***(W303)*** *myc-LoxP-nup145ΔGLFG myc-LoxP-nup57ΔGLFG HA-LoxP-nup100ΔGLFG*	14

Supplementary Table 1 (continued)

Strain code	Name	Relevant genotype	Source/Reference
SWY2980	*nup145ΔFG nup100ΔFG nsp1ΔFGΔFxFG*	***(W303)*** *myc-LoxP-nup145ΔGLFG HA-LoxP-nup100ΔGLFG Flag-LoxP-nsp1ΔFGΔFxFG*	14
SWY2922	*nsp1ΔFxFG-ΔFG*	***(W303)*** *Flag-LoxP-nsp1ΔFxFG-ΔFG*	14
SWY2801	*nup1ΔFxFG*	***(W303)*** *T7-LoxP-nup1ΔFxFG*	14
YV929	*ULP1-GFP mat a*	*Ulp1-GFP::HIS3MX*	Invitrogen
YV2049	*ULP1-GFP*	***(W303)*** *Ulp1-GFP::HIS3MX*	This study (g)
YV2052	*ULP1-GFP nup145ΔFG nup57ΔFG nup100ΔFG*	***(W303)*** *Ulp1-GFP::HIS3MX myc-LoxP-nup145ΔGLFG myc-LoxP-nup57ΔGLFG HA-LoxP-nup100ΔGLFG*	This study (g)
YV2050	*ULP1-GFP nup145ΔFG nup100ΔFG nsp1ΔFGΔFxFG*	***(W303)*** *Ulp1-GFP::HIS3MX myc-LoxP-nup145ΔGLFG HA-LoxP-nup100ΔGLFG Flag-LoxP-Nsp1ΔFGΔFxFG*	This study (g)
YV2069	*ULP1-GFP nsp1ΔFGΔFxFG*	***(W303)*** *Ulp1-GFP::HIS3MX Flag-LoxP-nsp1ΔFxFG-ΔFG*	This study (g)
YV2066	*ULP1-GFP nup1ΔFxFG*	***(W303)*** *Ulp1-GFP::HIS3MX T7-LoxP-nup1ΔFxFG*	This study (g)
YV1970	*HEK2-GFP*	*HEK2-GFP::HIS3MX*	Invitrogen

Homozygous and heterozygous deletion strains were obtained from the Euroscarf deletion collection (www.euroscarf.de). GFP-tagged strains were purchased from Invitrogen.

* obtained by transformation and/or successive crosses.
a. *HEK2* was deleted by homologous recombination with a cassette amplified from pFA6a-kanMX6.
b. *HEK2* was C-terminally tagged with 3 HA repeats by homologous recombination with a cassette amplified from pFA6a-3HA-kanMX6.
c. Segregant of a heterozygous diploid *ulp1::kanMX/ULP1*+ transformed with the *YCpLac22-HIS3-ulp1-333* construct.
d. Segregant of a heterozygous diploid *ubc9::kanMX/UBC9*+ transformed with the *pRS315-ubc9-1* construct.
e. Segregant of a heterozygous diploid *smt3::kanMX/SMT3*+ transformed with the *pRS316-SMT3* construct.
f. Segregant of a diploid obtained by mating *ulp1* and *SMT3 shuffle* strains, transforming with the *pYES2-LEU2-His-Flag-SMT3* construct and further growing on 5FOA to counterselect the *SMT3-URA3* plasmid.
g. *ULP1* was C-terminally tagged with GFP by homologous recombination with a cassette amplified from the *ULP1-GFP* strain (Invitrogen).

Supplementary Table 2: Plasmids used in this study

Name	Description	Source/Reference
pBXA	*for prot-A tagging*	15
pTH4	*trp1::HIS3 disruption fragment*	16
pTL7	*trp1::LEU2 disruption fragment*	16
pUL9	*ura3::LEU2 disruption fragment*	16
pGP564-ChromosomeII-152618-169095	*contains a genomic fragment encompassing the HEK2 gene*	Dharmacon yeast genomic tilling collection
pRS316-HEK2±500pb	*CEN/URA3/HEK2±500pb*	This study (a)
pRS316-HEK2-3HA	*CEN/URA3/HEK2-3HA (HEK2 natural promoter)*	This study (b)
pRS316-HEK2-pA	*CEN/URA3/HEK2-protA (HEK2 natural promoter)*	This study (c)
pET28b-HEK2-3HA-His6	*AmpR/HEK2-3HA-His6 (for recombinant protein production)*	This study (d)
pGEX-6p-1	*AmpR/TACprom-GST (for recombinant protein production)*	Addgene
pFA6a-kanMX	*for deletion*	17
pYEP96-6His-SMT3	*CEN/TRP1/CUP1prom-6His-SMT3*	1
pYEP96-LEU2-6His-SMT3	*CEN/LEU2/CUP1prom-6His-SMT3*	This study (e)
pYES2-His-Flag-SMT3	*2μ/URA3/GALprom-His-Flag-SMT3*	a gift from V. Géli
pYES2-LEU2-His-Flag-SMT3	*2μ/LEU2/GALprom-His-Flag-SMT3*	This study (f)
pFA6a-3HA-kanMX6	*for 3-HA tagging*	17
pRS315-*ubc9-1*	*CEN/LEU2/ubc9-1 (a thermosensitive ubc9 allele ; UBC9 natural promoter)*	This study (g)
YCpLac22-ulp1-333	*CEN/TRP1/ulp1-333 (thermosensitive ulp1 allele ; ULP1 natural promoter)*	8, 13
YCpLac111-ulp1-333	*CEN/LEU2/ulp1-333 (thermosensitive ulp1 allele ; ULP1 natural promoter)*	1
YCpLac22-HIS3-ulp1-333	*CEN/HIS3/ulp1-333 (thermosensitive ulp1 allele ; ULP1 natural promoter)*	This study (h)
pRS316-SMT3	*CEN/URA3/SMT3 (SMT3 natural promoter)*	This study (i)
pRS316-hek2-3HA K1-30R	*CEN/URA3/hek2-3HA K1-30R*	This study (j)
pRS316-hek2-3HA K1-7R	*CEN/URA3/hek2-3HA K1-7R*	This study (j)
pRS316-hek2-3HA K8-18R	*CEN/URA3/hek2-3HA K8-18R*	This study (j)
pRS316-hek2-3HA K19-30R	*CEN/URA3/hek2-3HA K19-30R*	This study (j)
pRS316-hek2-3HA K8-12R	*CEN/URA3/hek2-3HA K8-12R*	This study (j)
pRS316-hek2-3HA K13-18R	*CEN/URA3/hek2-3HA K13-18R*	This study (j)
pRS316-hek2-3HA K19-24R	*CEN/URA3/hek2-3HA K19-24R*	This study (j)
pRS316-hek2-3HA K25-30R	*CEN/URA3/hek2-3HA K25-30R*	This study (j)
pRS316-hek2-3HA K13-14R	*CEN/URA3/hek2-3HA K13-14R*	This study (j)
pRS316-hek2-3HA K15R	*CEN/URA3/hek2-3HA K15R*	This study (j)
pRS316-hek2-3HA K16-18R	*CEN/URA3/hek2-3HA K16-18R*	This study (j)
pRS316-hek2-3HA K25-26R	*CEN/URA3/hek2-3HA K25-26R*	This study (j)
pRS316-hek2-3HA K27-28R	*CEN/URA3/hek2-3HA K27-28R*	This study (j)
pRS316-hek2-3HA K29-30R	*CEN/URA3/hek2-3HA K29-30R*	This study (j)
pRS316-hek2-3HA K15R K29-30R	*CEN/URA3/hek2-3HA K15R K29-30R*	This study (j)
pET21b-His-UBC9	*AmpR/His-UBC9 (for recombinant protein production)*	18
pET11-His-UBA2	*AmpR/His-UBA2 (for recombinant protein production)*	18
pET-His-AOS1	*KanR/His-AOS1 (for recombinant protein production)*	18
pET21b-His-SMT3	*AmpR/His-SMT3 (for recombinant protein production)*	18
pET21b-His-SMT3 K11-15-19R	*AmpR/His-SMT3 K11-15-19R (for recombinant protein production)*	This study (k)

Supplementary Table 2 (continued)

a. A genomic fragment encompassing *HEK2* CDS ± 500bp was amplified from pGP564-ChromosomeII-152618-169095 and subcloned within pRS316.
b. A genomic fragment encompassing the *HEK2-3HA* allele was amplified from the *HEK2-3HA* strain and subcloned within pRS316.
c. The prot-A tag was amplified from pBXA and cloned in between AscI-PacI sites of pRS316-HEK2-3HA.
d. A PCR fragment encompassing *HEK2* CDS was amplified from pRS316-HEK2-3HA and cloned in between NcoI-XhoI sites of pET28b+.
e. The *TRP1* marker was swapped by homologous recombination with a disruption fragment from pTL7. This plasmid encodes a His-tagged version of Smt3 (yeast SUMO) under the control of the copper-inducible *CUP1* promoter.
f. The *URA3* marker was swapped by homologous recombination with a disruption fragment from pUL9. This plasmid encodes a His-Flag doubly-tagged version of Smt3 under the control of the galactose-inducible *GAL1/10* promoter.
g. A genomic fragment encompassing the *UBC9* complete CDS (+/-500bp) was PCR-amplified form yeast genomic DNA and cloned within pRS315. The *ubc9-1* point mutation[7] was then introduced by site directed mutagenesis using the QuickChange XL Site-Directed Mutagenesis Kit (Agilent).
h. The *TRP1* marker from *YCpLac22-ulp1-333* was swapped by homologous recombination with a disruption fragment from pTH4.
i. A genomic fragment encompassing the *SMT3* complete CDS (+/-500bp) was PCR-amplified form yeast genomic DNA and cloned within pRS316.
j. Synthetic genes encompassing *HEK2* sequences harboring stretches of lysines mutated to arginines were synthesized by ATG biosynthetics or Genecust. PCR-based techniques were used to combine *wt* and *KR HEK2* fragments to express the different HA-tagged chimeras under the control of *HEK2* natural promoter in the pRS316 backbone.
k. Generated by site directed mutagenesis of pET21b-His-SMT3 using the QuickChange XL Site-Directed Mutagenesis Kit (Agilent).

Supplementary Table 3: qPCR primers used in this study

NUP133-F	CGCCCAGGTGCATACTAACT
NUP133-R	AATGATAAGCCCTCCGGTTT
NUP170-F	TGTGGATCATTCTGCTCTGC
NUP170-R	CGCAAGCCAATTTCTTTAGC
NUP59-F	CACCACAGACAACCCAGATG
NUP59-R	AATTGCAAGTGTTGCTGCTG
NUP188-F	CACAACATTTGGAGCAATGG
NUP188-R	GGCACGTCTCAGGTAAAACC
NUP116-F	CCTTTGGTCAGGTGAATCGT
NUP116-R	TTTGCGTTAGCGTTTGATTG
NUP100-F	GGGATCTTGTCACCTTTGGA
NUP100-R	ATTAATGCCTTCGCCCTTTT
NSP1-F	CCCTTTCATTTGGTTCAGGA
NSP1-R	GCTGGTTTTGCTGGTTCATT
NUP57-F	CGGCAATAGCACTCAAAACA
NUP57-R	CCAAATAGGCCTCCCGTAGT
NUP1-F	CTCTGAGGGAAGTGCGAAAC
NUP1-R	CGAAAACGAGGGTTTAGCTG
NUP2-F	CGCAAGATGCAACCAAAGTA
NUP2-R	AAGCCACTTCGTCTTCCTCA
ASH1-F	ACGAAAAGTGGCAAGATGAG
ASH1-R	TGATAATTGGGTGACCTTGG
ACT1-F	ACGTTACCCAATTGAACACG
ACT1-R	AGAACAGGGTGTTCTTCTGG
rRNA 25S-F	AACGTCTATGCGAGTGTTTGG
rRNA 25S-R	TTCCTCTGGCTTCACCCTATT

Supplementary References

1. Bretes H*, et al*. Sumoylation of the THO complex regulates the biogenesis of a subset of mRNPs. *Nucleic Acids Res* **42**, 5043-5058 (2014).

2. Hasegawa Y, Irie K, Gerber AP. Distinct roles for Khd1p in the localization and expression of bud-localized mRNAs in yeast. *RNA* **14**, 2333-2347 (2008).

3. Kim Guisbert K, Duncan K, Li H, Guthrie C. Functional specificity of shuttling hnRNPs revealed by genome-wide analysis of their RNA binding profiles. *RNA* **11**, 383-393 (2005).

4. Li Z, Lee I, Moradi E, Hung NJ, Johnson AW, Marcotte EM. Rational extension of the ribosome biogenesis pathway using network-guided genetics. *PLoS Biol* **7**, e1000213 (2009).

5. Eisinger DP, Dick FA, Trumpower BL. Qsr1p, a 60S ribosomal subunit protein, is required for joining of 40S and 60S subunits. *Mol Cell Biol* **17**, 5136-5145 (1997).

6. Cridge AG*, et al*. Identifying eIF4E-binding protein translationally-controlled transcripts reveals links to mRNAs bound by specific PUF proteins. *Nucleic Acids Res* **38**, 8039-8050 (2010).

7. Schwarz SE, Matuschewski K, Liakopoulos D, Scheffner M, Jentsch S. The ubiquitin-like proteins SMT3 and SUMO-1 are conjugated by the UBC9 E2 enzyme. *Proc Natl Acad Sci U S A* **95**, 560-564 (1998).

8. Lewis A, Felberbaum R, Hochstrasser M. A nuclear envelope protein linking nuclear pore basket assembly, SUMO protease regulation, and mRNA surveillance. *J Cell Biol* **178**, 813-827 (2007).

9. Alber F*, et al*. The molecular architecture of the nuclear pore complex. *Nature* **450**, 695-701 (2007).

10. Winey M, Yarar D, Giddings TH, Jr., Mastronarde DN. Nuclear pore complex number and distribution throughout the Saccharomyces cerevisiae cell cycle by three-dimensional reconstruction from electron micrographs of nuclear envelopes. *Mol Biol Cell* **8**, 2119-2132 (1997).

11. Ghaemmaghami S*, et al*. Global analysis of protein expression in yeast. *Nature* **425**, 737-741 (2003).

12. Paquin N, Menade M, Poirier G, Donato D, Drouet E, Chartrand P. Local activation of yeast ASH1 mRNA translation through phosphorylation of Khd1p by the casein kinase Yck1p. *Mol Cell* **26**, 795-809 (2007).

13. Li SJ, Hochstrasser M. A new protease required for cell-cycle progression in yeast. *Nature* **398**, 246-251 (1999).

14. Strawn LA, Shen T, Shulga N, Goldfarb DS, Wente SR. Minimal nuclear pore complexes define FG repeat domains essential for transport. *Nat Cell Biol* **6**, 197-206 (2004).

15. Rout MP, Aitchison JD, Suprapto A, Hjertaas K, Zhao Y, Chait BT. The yeast nuclear pore complex: composition, architecture, and transport mechanism. *J Cell Biol* **148**, 635-651 (2000).

16. Cross FR. 'Marker swap' plasmids: convenient tools for budding yeast molecular genetics. *Yeast* **13**, 647-653 (1997).

17. Longtine MS*, et al*. Additional modules for versatile and economical PCR-based gene deletion and modification in Saccharomyces cerevisiae. *Yeast* **14**, 953-961 (1998).

18. Johnson ES, Blobel G. Ubc9p is the conjugating enzyme for the ubiquitin-like protein Smt3p. *J Biol Chem* **272**, 26799-26802 (1997).
